# Astrocytes Are More Vulnerable than Neurons to Silicon Dioxide Nanoparticle Toxicity in Vitro

**DOI:** 10.3390/toxics8030051

**Published:** 2020-07-29

**Authors:** Jorge Humberto Limón-Pacheco, Natalie Jiménez-Barrios, Alejandro Déciga-Alcaraz, Adriana Martínez-Cuazitl, Mónica Maribel Mata-Miranda, Gustavo Jesús Vázquez-Zapién, Jose Pedraza-Chaverri, Yolanda Irasema Chirino, Marisol Orozco-Ibarra

**Affiliations:** 1 Laboratorio de Biología Celular y Tisular, Escuela Militar de Medicina, Centro Militar de Ciencias de la Salud, Secretaría de la Defensa Nacional, Cerrada de Palomas S/N, Lomas de San Isidro, Alcaldía Miguel Hidalgo, Ciudad de Mexico 11200, Mexico; akelason1977@gmail.com (J.H.L.-P.); adyta0@hotmail.com (A.M.-C.); mmcmaribel@gmail.com (M.M.M.-M.); 2 Laboratorio de Neurobiología Molecular y Celular, Instituto Nacional de Neurología y Neurocirugía, Manuel Velasco Suárez, Av. Insurgentes Sur # 3877, La Fama, Alcaldía Tlalpan, Ciudad de Mexico 14269, Mexico; nataliej.k.barrios@gmail.com; 3 Laboratorio de Carcinogénesis y Toxicología, Unidad de Biomedicina, Facultad de Estudios Superiores Iztacala, Universidad Nacional Autónoma de Mexico, Avenida de los Barrios # 1, Los Reyes Iztacala, Tlalnepantla, Estado de Mexico 54090, Mexico; alejandro.deciga01@gmail.com (A.D.-A.); irasemachirino@gmail.com (Y.I.C.); 4 Laboratorio de Embriología, Escuela Militar de Medicina, Centro Militar de Ciencias de la Salud, Secretaría de la Defensa Nacional, Cerrada de Palomas S/N, Lomas de San Isidro, Alcaldía Miguel Hidalgo, Ciudad de Mexico 11200, Mexico; gus1202@hotmail.com; 5 Departamento de Biología, Facultad de Química, Universidad Nacional Autónoma de Mexico, Ciudad Universitaria, Alcaldía Coyoacán, Ciudad de Mexico 04510, Mexico; pedraza@unam.mx

**Keywords:** cerebellar granule neurons, nanoparticle exposure, neurotoxic effects, secondary astrocytes, silicon dioxide

## Abstract

Some studies have shown that silicon dioxide nanoparticles (SiO_2_-NPs) can reach different regions of the brain and cause toxicity; however, the consequences of SiO_2_-NPs exposure on the diverse brain cell lineages is limited. We aimed to investigate the neurotoxic effects of SiO_2_-NP (0–100 µg/mL) on rat astrocyte-rich cultures or neuron-rich cultures using scanning electron microscopy, Attenuated Total Reflection-Fourier Transform Infrared spectroscopy (ATR-FTIR), FTIR microspectroscopy mapping (IQ mapping), and cell viability tests. SiO_2_-NPs were amorphous particles and aggregated in saline and culture media. Both astrocytes and neurons treated with SiO_2_-NPs showed alterations in cell morphology and changes in the IR spectral regions corresponding to nucleic acids, proteins, and lipids. The analysis by the second derivative revealed a significant decrease in the signal of the amide I (α-helix, parallel β-strand, and random coil) at the concentration of 10 µg/mL in astrocytes but not in neurons. IQ mapping confirmed changes in nucleic acids, proteins, and lipids in astrocytes; cell death was higher in astrocytes than in neurons (10–100 µg/mL). We conclude that astrocytes were more vulnerable than neurons to SiO_2_-NPs toxicity. Therefore, the evaluation of human exposure to SiO_2_-NPs and possible neurotoxic effects must be followed up.

## 1. Introduction

Silicon dioxide (SiO_2_), labeled as E551 in Europe, has been authorized as a food additive for more than two decades. However, as nanotechnology develops, the use of SiO_2_ in nanometric size has increased [1] in the cosmetic and pharmaceutical industry due to its anti-caking properties. The growing potential exposure due to multiple sources in the environment has raised a global concern regarding the safety and possible adverse health effects of SiO_2_ nanoparticles (SiO_2_-NPs) by inhalation and oral exposure. Indeed, the European Food Safety Authority Panel on Food Additives and Nutrient Sources added to Food called to reassess the toxicity of SiO_2_ as a food additive in 2018 [2]. Mainly, crystalline SiO_2_ has been classified as a carcinogen to humans by the International Agency of Research in Cancer, and then, the possibility of adverse effects of amorphous SiO_2_ is under investigation. Some studies indicate that amorphous SiO_2_-NPs can be as reactive as crystalline particles, which may result in adverse health effects [3,4].

Additionally, SiO_2_-NPs are cytotoxic, genotoxic, immunotoxic, and induce cell death in several in vivo and in vitro experimental models [5]. SiO_2_-NPs can translocate to the bloodstream after inhalation [5,6]; even more, they can cross the placenta and reach the fetal brain and the liver as demonstrated in a mouse model [7]. Indeed, subchronic administration by nasal instillation of SiO_2_-NPs in adult rats resulted in NPs accumulation in the olfactory bulb, the striatum, the hippocampus, the brain stem, the cerebellum, and the frontal cortex [8], and some studies indicate an association to oxidative stress and inflammation [9,10]. Moreover, SiO_2_-NPs could disturb the structure and function of the blood–brain barrier by inducing inflammation through reactive oxygen species (ROS) generation and by activation of Rho-kinase/JNK signaling pathways [11]. Moreover, as NPs can reach the brain in different regions [7,8] and based on the diversity of cell lineages, a differential vulnerability could be expected, for instance in neurons and astrocytes, two of the most relevant cell types for brain physiology. On the other hand, several techniques have been used to characterize the size, crystal structure, elemental composition, and other physical properties of NPs [12]. Fourier Transform Infrared spectroscopy (FTIR) is a technique based on the measurement of the absorption of electromagnetic radiation with wavelengths within the mid-infrared region (IR) (4000–400 cm^−1^) [12] that identifies variations in functional groups through the measure of the vibration and rotation of molecules influenced by IR [13]. The IR spectrum analysis has been used mainly to analyze pure samples, but biological interactions with SiO_2_-NPs can also be analyzed because molecular interactions produce a specific molecular fingerprint [12,14]. Thus, the present work aimed to study the neurotoxic effect of SiO_2_-NPs on rat astrocyte-rich or rat neuron-rich cultures by Attenuated Total Reflection spectroscopy (ATR-FTIR) and FTIR microspectroscopy mapping (IQ mapping) to characterize biomolecular fingerprints and cellular susceptibility determining cell morphology and cell viability.

## 2. Materials and Methods

### 2.1. Reagents

We obtained SiO_2_-NPs catalog number 637,238 and most of the used reagents from Sigma-Aldrich (St. Louis, MO, USA). The SiO_2_-NPs used in this study were amorphous, non-crystalline, sized less than 100 nm, and tend to agglomerate. All other reagents were of analytical grade and commercially available.

### 2.2. Bioethics

Rats used in this study were maintained and manipulated according the Norma Oficial Mexicana NOM-062-ZOO-1999, and a local Committee on Research Ethics approved the procedures to isolate astrocytes and neurons from neonatal rats (Approbation number at Instituto Nacional de Neurología y Neurocirugía: 19/17, date: 4 July 2017).

### 2.3. Scanning Electron Microscopy (SEM) and Size Distribution

The original size of SiO_2_-NPs was 10–20 nm, according to the Sigma-Aldrich data sheet (Cat. No.637238, St. Louis, MO, USA). Then, the shape was analyzed using a JSM-7800F Schottky Field Emission Scanning Electron Microscope (JEOL Ltd., Tokyo, Japan) with 3.0 kV and 26 pixels/nm^2^. The size distribution was measured using ImageJ software version 1.52a (1997-2018, National Institutes of Health, Bethesda, MD, USA). Briefly, the pixel size in each image was adjusted using the scale bar. The area of at least forty particles was registered. The area-equivalent average diameters of all the reported particles generated a number-based, cumulative particle size distribution.

### 2.4. SiO_2_-NPs Hydrodynamic Size, Polydispersion Index and Zeta (ζ) Potential

For hydrodynamic size and ζ-potential, 1 mg of SiO_2_-NPs was suspended in 1 mL of Basal Medium Eagle (BME) or Dulbecco’s Modified Eagle Medium (DMEM) cell culture, and both were supplemented with 10% of fetal bovine serum (FBS) and 1% of antibiotic-antifungal GibcoTM, (Thermo Fisher Scientific, Waltham, MA, USA). Then, the suspension was sonicated at 60 Hz for 30 min. Immediately, the stocks were diluted in a ratio of 1:100 in cell culture medium for hydrodynamic size measured by dynamic light scattering and for polydispersion index and ζ-potential using a Zeta Plus Analyzer (Brookhaven Instruments, Holtsville, NY, USA). 

### 2.5. Secondary Culture of Rat Cerebellar Astrocytes

We isolated rat astrocyte-rich secondary cultures following a previously reported protocol with minor modifications [15]. In brief, astrocytes were isolated from the cerebellum of 7-9-days-old male/female Wistar rats. Cerebellum was suspended in Tyrode solution and disintegrated by passing through a 21G × 32 mm needle three times and then through a 27G × 13 mm needle three more times. Then, it was incubated 15 min at 37 °C and centrifuged at 500× *g* to pellet the resulting cerebellar dissociated cells, which were suspended in DMEM medium supplemented with 10% FBS and antibiotic-antimycotic GibcoTM (Thermo Fisher Scientific, Waltham, MA, USA). Then, the mixed cerebellar cells were plated in T75 flasks previously treated with 10 µg/mL poly-D-lysine, and the cell culture medium was changed every four days. After 28 days, when astrocytes were confluent, the T75 flasks were placed on an orbital shaker incubator at 180 rpm for 24 h to remove microglia and other glial cells. Then, DMEM was discarded, and astrocytes were washed and incubated with 0.5% Trypsin-EDTA for 5 min at 37 °C. After astrocytes were detached from the culture flask, they were suspended in fresh supplemented DMEM and reseeded at 16 × 10^3^ cells/cm^2^ in 24-well plates to obtain astrocyte-rich cultures. The identity of these cultures was confirmed through a GFAP immunodetection (Figure S1A). 

### 2.6. Primary Culture of Rat Cerebellar Granule Neurons

We isolated rat cerebellar granule neuron-rich cultures from the cerebellum of 7-9-days-old male/female Wistar rats following standardized protocols [16]. Briefly, the cerebellum was removed, cut within small pieces, and incubated with 0.25% trypsin solution by 10 min at 37 °C. Then, 0.08% DNase I was added to degrade the free DNA, and tissue was dissociated by filtration through a sterile polyester mesh of 50 µm pore size. Cells were suspended in BME supplemented with 10% BSA, 2 mM L-glutamine, and antibiotic-antifungal GibcoTM (Thermo Fisher Scientific, Waltham, MA, USA). A total of 300 × 10^3^ cells/cm^2^ were seeded in 24-well plates previously coated with 5 µg/mL poly-L-lysine. Cytosine arabinose (10 µM) was added after 24 h, and glucose (5 mM) after four days from seeding. According to a morphological examination followed by immunodetection studies using antibodies against the glial fibrillary acidic protein (GFAP, Millipore Co, Burlington, MA, USA), microtubule-associated protein 2 (MAP-2, Millipore Co, Burlington, MA, USA), and 4′,6-diamidino-2-phenylindole (DAPI, Thermo Fisher Scientific, Waltham, MA, USA), glial, neuronal, and nuclear markers, respectively, about 2% of glial cells are present in our culture conditions (Figure S1B). 

### 2.7. Concentration-Response Analysis to SiO_2_-NPs

Astrocyte-rich cultures were exposed to SiO_2_-NPs at day two after reseeding, when reached at least 80% of confluence, whereas neurons were exposed to SiO_2_-NPs after 9 days of culture. A stock solution of 1 mg/mL SiO_2_-NPs was done in DMEM medium or BME (for astrocytes or neurons, respectively) and then was sonicated at 37 °C for 30 min at 50/60 Hz in a 2200 ultrasonic Cleaner, (Branson Ultrasonics, Danbury, CT, USA). Cell cultures were exposed to 0, 1, 10, 25, 50, and 100 µg/mL of SiO_2_-NPs by 24 h in fresh DMEM for astrocytes and conditioned medium for neurons. Bright-field images were obtained for each condition through a Nikon microscope with NIS-Elements software (Nikon Instruments Inc., Melville, NY, USA).

### 2.8. ATR-FTIR Spectroscopy

FTIR spectral analysis was conducted in the spectral range between 4000–400 cm^−1^ using a Jasco FT/IR-6600 spectrometer (Jasco Corporation, Tokyo, Japan) in the ATR-FTIR sampling mode. For SiO_2_-NPs FTIR analysis, a small amount of dry or saline-suspended SiO_2_-NPs was deposited onto the surface of the ATR crystal and dried at room temperature for 12 min to eliminate water excess. Control or treated cells were washed twice with saline (0.9% NaCl) and suspended in 50 µL of fresh saline solution. A sample of 4 μL of cell suspension was deposited onto the surface of the ATR-FTIR crystal and air-dried, as mentioned before. The spectrum for each sample was the average of 240 data acquisitions. Once all FTIR spectra were acquired (raw spectra), a standard normal variate normalization process was applied by using UNSCRAMBLER X, Version 10.3 (CAMO Analytics, Montclair, NJ, USA, 2011). After, the calculation of the second derivative of each spectrum was performed through the Savitzky–Golay algorithm, which uses a fitting successive subset of adjacent data points with a small degree polynomial by linear least squares as has been reported [17]. The spectral data were plotted using Origin (Pro), Version 6.0 (OriginLab Corporation, Northampton, MA, USA, 1999).

### 2.9. FTIR Microspectroscopy Mapping (IQ Mapping)

Biochemical changes in lipids, proteins, and nucleic acids in astrocyte-rich cultures and neuron-rich cultures exposed to SiO_2_-NPs were studied by FTIR microspectroscopy mapping, as previously reported with minor modifications [18]. FTIR microspectroscopy produces maps of color derived either directly from distinct spectral parameters in the sample, such as the functional groups of biomolecules, from multiple points in a specified area in a biological specimen. Biochemical images were obtained using the automated mapping of multiple points (IQ mapping) function of the FTIR microscope Jasco IRT-5200 coupled to a Jasco FT/IR-6600 spectrometer (Jasco Corporation, Tokyo, Japan) and a liquid nitrogen-cooled Mercury, Cadmium, Tellurium detector. In this function, the complete system automatically scans a specified area of a cell sample, collecting a full spectrum of each point without moving the sample with a 32× Cassegrain objective. Briefly, a sample of 4 µL of the suspended cells control or treated was deposited on a low reflective glass slide and dispersed to form a monolayer of cells and air-dried at room temperature for 12 min to remove water excess. The acquisition of images was performed in the reflectance mode with a spectral resolution of 4 cm^−1^ 60 scans and the spectral range of 400–4000 cm^−1^. The spectra were baseline corrected to compensate for atmospheric changes and ensure that the probe was free of cellular material using a free cells field in the low reflective glass slide as a background. The analyzed regions of the spectra were 989–1185 cm^−1^ for nucleic acids, 1593–1712 cm^−1^ for secondary structure of proteins, and 2830–2945 cm^−1^ for lipids. The analysis of each spectral region was represented as a tridimensional area, corresponding to the density distribution of the specimen’s biochemical components. The color scales for the maps were adjusted employing the same color for each biochemical component represented and taking care that scales were comparable between the experimental groups.

### 2.10. Cell Viability Tests

The calcein-AM uptake (Thermo Fisher Scientific, Waltham, MA, USA) and the 3-(4,5-dimethylthiazol-2-yl)-2,5-diphenyltetrazolium bromide (MTT) reduction assays were used to evaluate the astrocytes and neurons viability. Living neurons uptake the cell-permeant dye calcein-AM and emit green fluorescence after its hydrolysis by the activity of cytoplasmic esterases. MTT is transformed into formazan blue by the activity of mitochondrial dehydrogenases, and absorbance has been reported directly proportional to the number of viable cells [19]. For calcein-AM uptake, cells were washed three times with a Tyrode solution pH = 7.4. Calcein-AM (2.5 µg/mL) solution was immediately added, and cells were incubated for 30 min at 37 °C and 5% CO_2_ in darkness. The cells were washed three more times with Tyrode solution, and fluorescence was measured at 485 nm excitation and 585 nm emission in a multimodal microplate reader (BioTek Instruments, Winooski, VT, USA). The increase in fluorescence indicates the calcein-AM uptake by the cells. For MTT reduction assay, a solution of 1 mg/mL MTT in phosphate buffer was prepared. This solution was diluted with either BME or DMEM medium in a proportion of 500 µL of medium per 250 µL (2:1) of MTT per well. After the MTT addition, the culture plate was incubated for one hour at 37 °C, and then the remaining MTT was removed. The formazan crystals precipitated in the wells were dissolved with 500 µL of isopropanol. The formazan absorbance was quantified at 570 nm in a multimodal microplate reader (BioTek Instruments, Winooski, VT, USA). Data were expressed as the percentage of calcein-AM uptake or MTT reduction in control wells.

### 2.11. Statistical Analysis

All the experiments were independently performed at least three times with three technical replicates. The results were expressed as mean ± SEM. Statistical significance was determined by one-way ANOVA followed by Tukey’s *post hoc* test or by two-way ANOVA followed by Bonferroni’s *post hoc* test, as appropriate, using the GraphPad Prism 6.0 software (San Diego CA, USA, 2012). Statistical difference was considered when the value of *p* < 0.05.

## 3. Results

### 3.1. FTIR and SEM Characterization of SiO_2_-NPs

**Figure 1:**
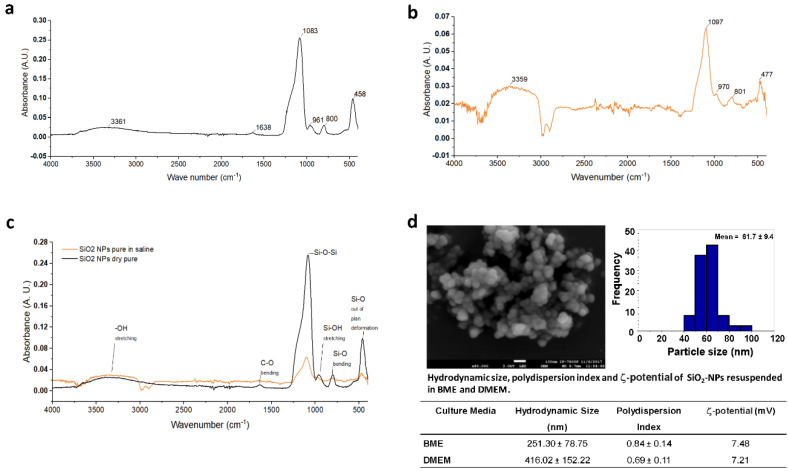
Characterization of SiO_2_-NPs by ATR-FTIR spectroscopy, scanning electron microscopy, and ζ-potential. (**a**) An infrared spectrum of SiO_2_-NPs as pure powder. IR spectral bands correspond to 458 cm^−1^ for Si-O out of plane deformation; 800 cm^−1^ for Si-O bending, 961 cm^−1^ for Si-OH stretching, 1083 cm^−1^ for Si-O-Si asymmetric stretching, 1638 cm^−1^ for C-O bending, 3361 cm^−1^ of -OH. (**b**) An infrared spectrum of SiO_2_-NPs suspended in saline solution. (**c**) Comparison of spectral bands of pure powder SiO_2_-NPs vs. suspended in saline. (**d**) Representative image of the scanning electron microscopy and ζ-potential values of SiO_2_-NPs suspended in BME and DMEM. The SEM image shown corresponds to 80,000×, scale bar 100 nm.

When nanoparticles are suspended in any medium, the properties of the material get modified to a great extent [20]. Due to this nanoparticles’ property, we analyzed by ATR-FTIR the spectral properties of the pure powder and the one suspended in 0.9% NaCl SiO_2_-NPs. Selection of NaCl 0.9% as a resuspension media obey the priority of avoiding interference or noisy signal in spectral regions evaluated, and because after exposure to concentrations of SiO_2_-NPs, we washed, collected, and stored cells in NaCl 0.9%. The analysis by FTIR of SiO_2_-NPs dry powder showed a spectral band region of 400–1700 cm^−1^ (Figure 1a). The absorption bands observed correspond to 458 cm^−1^ to Si-O out of plane deformation, 800 cm^−1^ to Si-O bending, 961 cm^−1^ to Si-OH stretching, 1083 cm^−1^ to Si-O-Si asymmetric stretching (Figure 1a,c). This set of spectral bands is characteristic of SiO_2_ and is similar to the spectral bands available in the PubChem Database and SpectraBase for the pure compound. Bands of 1638 cm^−1^ for C-O bending and 3361 cm^−1^ for -OH stretching was also detected (Figure 1a,c). When SiO_2_-NPs were suspended in saline solution, spectral bands detected were 477 cm^−1^ for Si-O out of plane deformation, 801 cm^−1^ for Si-O bending, 970 cm^−1^ for Si-OH stretching, 1097 cm^−1^ for Si-O-Si asymmetric stretching (Figure 1b). For the -OH stretching band, the spectral band corresponded to 3359 cm^−1^. Under this condition, no absorption band for C-O bending was detected (Figure 1c). Moreover, as in the case of the pure powder of SiO_2_-NPs suspended in saline solution, the band corresponding to Si-C stretching was not detected (Figure 1b,c). The spectral band of 1083 cm^−1^ for Si-O-Si asymmetric stretching is the most characteristic band for SiO_2_-NPs.

On the other hand, according to the manufacturer, the SiO_2_-NPs used in the present study have an original size of 10–20 nm determined by Brunauer–Emmett–Teller (BET) surface area analysis. In this study, the analysis of SiO_2_-NPs by SEM displayed an amorphous shape and sized below 100 nm (Figure 1d). When the SiO_2_-NPs were suspended in culture media, the hydrodynamic size of SiO_2_-NPs dispersed in BME and DMEM were of 251.3 ± 78.75 nm and 416 ± 152 nm, respectively. The ζ-potential of SiO_2_-NPs in BME was 7.48 mV and in DMEM was 7.21 mV (Figure 1d). Finally, the polydispersion index was 0.84 ± 0.14 for SiO_2_-NPs in BME and 0.69 ± 0.11 for SiO_2_-NPs dispersed in DMEM.

### 3.2. Morphological Changes of Rat Cerebellar Astrocytes and Neurons Exposed to SiO_2_-NPs

**Figure 2:**
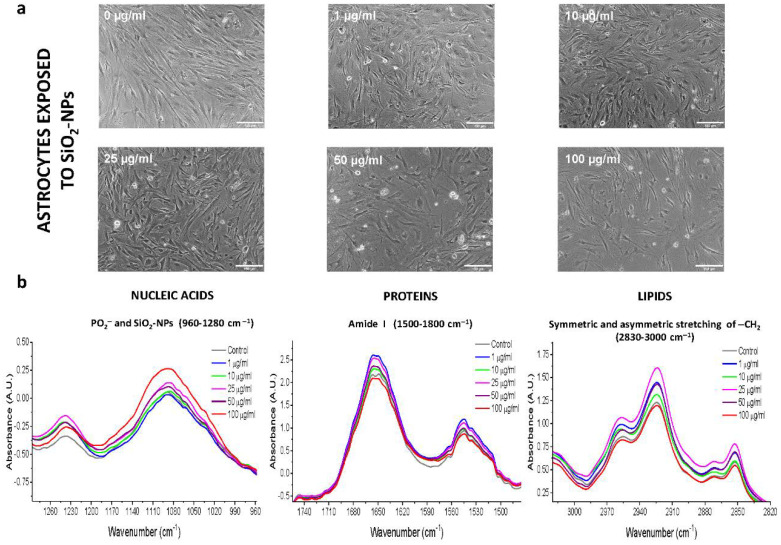
Morphological changes and normalized IR spectra of primary cultures of astrocytes exposed to SiO_2_-NPs. (**a**) Astrocyte morphology after 24 h of SiO_2_-NPs exposure. Images were obtained by light microscopy. Each treatment was performed at least in triplicate (*n* = 3) with three technical replicates. Subtle morphological changes compared to control culture are appreciated at 1, 10, and 25 µg/mL SiO_2_-NPs. At 50 µg/mL and 100 µg/mL SiO_2_-NPs, the size and amount of the astrocytes decreased markedly in comparison with the control. Scale bar: 150 µm. (**b**) Normalized IR spectra of astrocytes exposed to SiO_2_-NPs. The spectral region of 960–1280 cm^−1^ corresponds to PO_2_- (nucleic acids) and SiO_2_-NPs; 1500–1800 cm^−1^ is the spectral region that contains the amide I spectral bands and representative bands of the secondary protein structure; 2830–3000 cm^−1^ includes the spectral bands of symmetric lipid CH_2_ and asymmetric CH_2_. The spectral images are representative of four independent experiments performed with each treatment, with three technical replicates.

**Figure 3:**
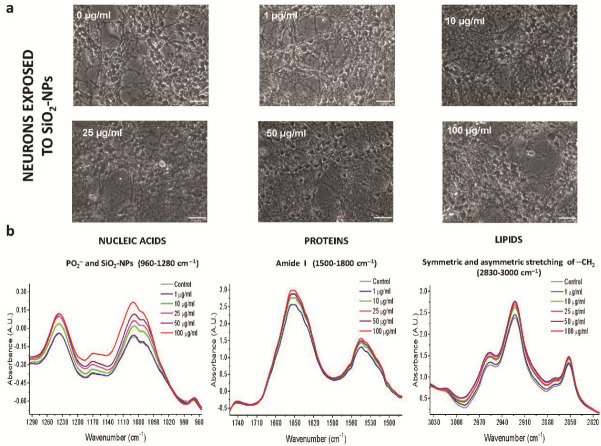
Morphological changes and normalized IR spectra of the primary cultures of neurons exposed to SiO_2_-NPs. (**a**) Neuron morphology after 24 h of SiO_2_-NPs exposure. Images were obtained by light microscopy. Each treatment was performed at least in triplicate (*n* = 3) with three technical replicates. There are no morphological changes in the concentrations of 0–25 µg/mL, but at 50 and 100 µg/mL, there are clear morphological changes such as a reduced cell volume and a decrease in the amount of cells. Scale bar: 150 µm. (**b**) Normalized IR spectra of neurons exposed to SiO_2_-NPs. The spectral region of 960–1280 cm^−1^ corresponds to PO_2_- (nucleic acids) and SiO_2_-NPs, 1500–1800 cm^−1^ to spectral region that contains the amide I spectral bands and representative bands of the secondary protein structure, and 2830–3000 cm^−1^ includes the spectral bands of symmetric lipid CH_2_ and asymmetric CH_2_. The spectral images are representative of four independent experiments performed with each treatment, with three technical replicates.

Control astrocytes possessed branching processes or long and thin unbranched processes, also showed flat and epithelioid morphology, arranged in monoculture, with scarce or null syncytium formed with other astrocytes (Figure 2a). After exposure to concentrations of 1, 10, and 25 µg/mL SiO_2_-NPs, astrocytes showed subtle morphological differences compared to control. However, astrocytes treated with 50 µg/mL SiO_2_-NPs showed marked changes in morphology, such as very thin, long, and scarce developed ramifications and small soma. Exposure to 100 µg/mL SiO_2_-NPs resulted in a markedly decreased size number of astrocytes compared with the control (Figure 2a). Meanwhile, control neurons showed long and abundant neurites, ramified and extensive (Figure 3a). In the neurons exposed to 10 µg/mL SiO_2_-NPs, small soma size and poor development of neurites were observed. From this concentration, loss of network conformation formed by the ramifications and thin and shortened cellular extensions were observed (Figure 3a).

### 3.3. ATR-FTIR Spectra of Rat Cerebellar Astrocytes and Neurons Exposed to SiO_2_-NPs

Analysis of biological samples through FTIR is possible thanks to specific regions of the IR spectrum that represent a biochemical fingerprint of the structure and function of specific biomolecules of interest in cellular specimens. In our study, we considered the fingerprint region of SiO_2_-NPs 600–1450 cm^−1^ observed previously (Figure 1a–c), which also includes nucleic acids and carbohydrates. Proteins are represented by the amide I and amide II region of 1500–1700 cm^−1^ and lipids by the higher region of 2550–3500 cm^−1^ associated with stretching vibrations of –CH_2_ [14]. A representative normalized IR spectrum for each experimental condition is shown in Figure S2, where we observed slight differences in the value of the normalized IR spectral band corresponding to −CH_2_ region of lipids, amide I region of proteins, and the PO_2_^−^ region of nucleic acids and carbohydrates between SiO_2_-NPs concentrations. A summary of the main IR spectral bands detected for astrocytes and neurons is presented in Supplementary Table S1.

Next, we analyzed the normalized IR spectrum and its absorbance in spectral bands of the nucleic acids, protein, and lipid regions (Figure 2b and Figure 3b). In astrocytes, the changes in the IR spectral bands and the absorbance were independent of the increase in the concentration of SiO_2_-NPs for the three spectral regions analyzed (Figure 2b). We found a significant difference in the absorbance in the PO_2_^−^ symmetric region at 1090 cm^−1^ (*p* = 0.025, *F*_(5,42)_ *=* 2.888, Figure 2b); and the amide I at 1650 cm^−1^ of protein region (*p* = 0.0409, *F*_(5,42)_ = 2.567). In the amide II region, no differences were detected (Figure 2b). No differences were observed in the lipid region (C=O, 1744 cm^−1^; −CH_2_ symmetric 2852 cm^−1^ and asymmetric 2923 cm^−1^). In the case of neurons, there were changes in the IR spectral band and absorbance in the nucleic acids, protein, and lipid regions (Figure 3b). The difference in the spectral band corresponding to the nucleic acids asymmetric PO_2_- at 1237 cm^−1^ was detected (*p* = 0.0015, *F*_(5,60)_ = 4.510) without significant changes in the absorbance (Figure 3b). No changes were observed in the spectral region of amide I at 1652 cm^−1^, amide II at 1545 cm^−1^ (Figure 3b), or C=O at 1740 cm^−1^ to lipids. The absorbance in the asymmetric -CH_2_ lipid region at 2923 cm^−1^ was different among concentrations (*p* = 0.010, *F*_(5,60)_ = 3.318), but no changes in the IR spectral band were observed. Taken together, all changes described in these spectral bands’ positions and absorbances indicated the biological interaction between SiO_2_-NPs and biochemical components of astrocytes and neurons.

### 3.4. Second Derivative Analyses of Spectral Bands

FTIR spectra of biological samples require a more in-depth examination because of their biochemical and molecular complexity, and changes in the absorbance of spectral bands of interest could be underestimated [21]. For that reason, the second derivative was used to analyze spectral bands that potentially overlapped in the IR spectrum of each SiO_2_-NPs concentration. Bands of particular interest due to their biological importance considered in this study were 1083 cm^−1^ to examine the incorporation of SiO_2_-NPs in rat astrocytes and neurons in agreement with the spectral fingerprint of the SiO_2_-NPs previously presented; 1090 cm^−1^ to evaluate changes in symmetric stretching PO_2_^−^ of sugar rings of nucleic acids, mainly DNA, and as an indicator of cell death [22]; and 1654 cm^−1^, 1639 cm^−1^, and 1627 cm^−1^ as indicators of changes in the proteins secondary structure corresponding to alpha-helix, random coil, and parallel beta-strand, respectively [23]. Spectral bands of 2852 cm^−1^ and 2923 cm^−1^ are representative of symmetric and asymmetric stretching of −CH_2_ of lipids, respectively, and have been previously considered as biomarkers of cell death [22]. A summary of the values obtained through the second derivative analysis is shown in Table 1.

### 3.5. Second Derivative Analyses of the Nucleic Acid Spectral Region

**Figure 4:**
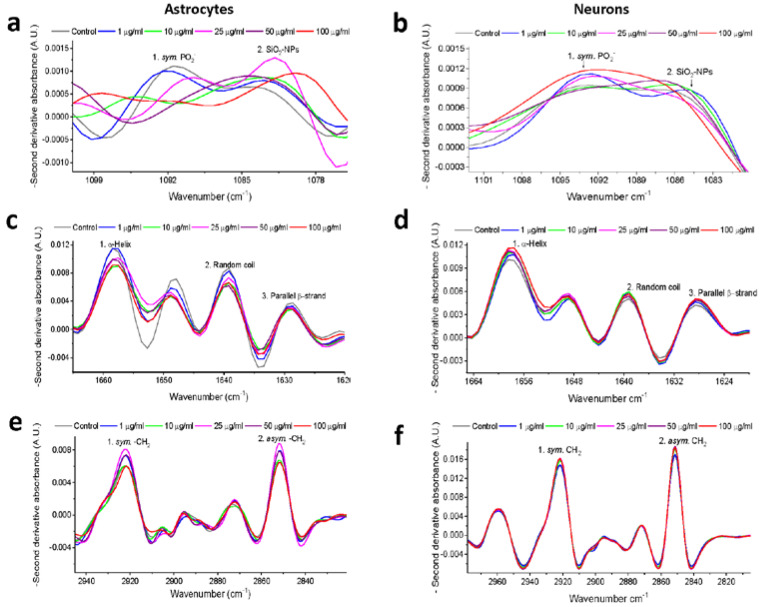
Second derivative spectra analysis of nucleic acids, amide I and lipid regions after SiO_2_-NPs exposure. Nucleic acids correspond to symmetric PO_2_^−^ at 1090 cm^−1^, and SiO_2_-NPs correspond to 1083 cm^−1^ spectral band in (**a**) astrocytes and (**b**) neurons. The secondary structure of proteins corresponds to the amide I region, which includes the 1627 cm^−1^ for parallel β-strand, 1639 cm^−1^ for random coil, and 1654 cm^−1^ for alpha-helix in (**c**) astrocytes and (**d**) neurons. Lipids correspond to 1852 cm^−1^ for symmetric -CH_2_ and 2923 cm^−1^ for asymmetric -CH_2_ in (**e**) astrocytes and (**f**) neurons.

We found significant changes in rat astrocytes treated with SiO_2_-NPs at the spectral bands of 1083 cm^−1^ (*p* = 0.0177, *F*_(5,42)_ = 3.112) and 1090 cm^−1^ (*p* = 0.0211, *F*_(5,42)_ = 2.996), and in both cases, the lowest signal was observed at 10 µg/mL (Tukey’s *post hoc* test, *p* < 0.05, Table 1). A biphasic behavior was observed as SiO_2_-NPs concentration increased (Figure 4a). In contrast, no changes were detected in neurons at the 1083 cm^−1^ (*p* = 0.595, *F*_(5,60)_ = 0.74) and 1090 cm^−1^ bands (Figure 4b, *p* = 0.540, *F*_(5,60)_ = 0.8198).

### 3.6. Second Derivative Analyses of the Protein Spectral Region

In rat astrocytes, SiO_2_-NPs produced significant differences in spectral bands at 1627 cm^−1^ (*p* = 0.0167, *F*_(5,42)_ = 3.149), 1639 cm^−1^ (*p* = 0.0241, *F*_(5,42)_ = 2.910), and 1654 cm^−1^ (*p* = 0.0246, *F*_(5,42)_ = 2.896), which are related to protein secondary structure (Figure 4c). The lowest signal for these spectral bands was observed at 10 µg/mL (Tukey’s *post hoc* test, *p* < 0.05, Table 1). In contrast, in neurons, no significant difference in those spectral bands was observed (Figure 4d).

### 3.7. Second Derivative Analyses of the Lipid Spectral Region

In astrocytes exposed to SiO_2_-NPs, we confirmed the biphasic behavior observed in the normalized IR spectra (Figure 4e), in the spectral band of 2852 cm^−1^, with the lowest signal detected at 10 µg/mL SiO_2_-NPs (*p* = 0.0452, *F*_(5,42)_ = 2.503). In neurons, this spectral band increases with concentration; however, no differences were observed (Figure 4f), and it was similar with the spectral band of 2923 cm^−1^ of asymmetric -CH_2_, and significant differences were observed in astrocytes (*p* = 0.0292, *F*_(5,42)_=2.786), with the lowest peak at the concentration of 10 µg/mL SiO_2_-NPs (*p* < 0.05, Table 1). In contrast, there were no observable differences in neurons at the spectral band of 2923 cm^−1^ under our experimental conditions. At this point, our data suggest a differential sensitivity between astrocytes and neurons to the SiO_2_-NPs toxicity (Table 1).

### 3.8. IQ mapping of Nucleic Acid Region 989–1185 cm^−1^ in Astrocytes and Neurons after Exposure to SiO_2_-NPs

**Figure 5:**
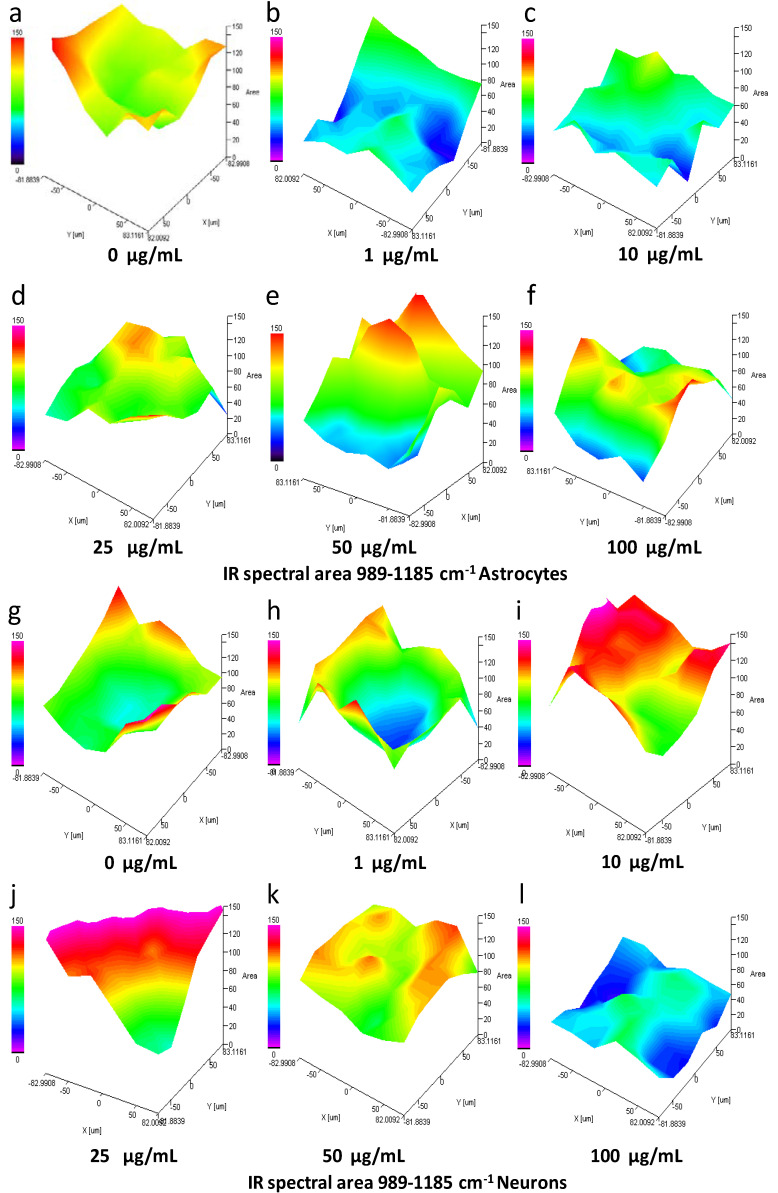
Spectral area analysis by IQ mapping of 989–1185 cm^−1^ region of astrocytes and neurons exposed to SiO_2_-NPs. The spectral region of 989–1185 cm^−1^ that includes specific spectral bands related to nucleic acids is presented for astrocytes (**a**–**f**) and neurons (**g**–**l**). A sample of cells treated with SiO_2_-NPs for 24 h was dispersed on a gold-coated slide. The three-dimensional image is presented in the x, y, and z axes, and the area presented as a gradient of color indicates a decrease (from blue) or increase (red) in the spectral signal. For astrocytes, a decrease in the signal was seen at 1 µg/mL (**b**) and 10 µg/mL (**c**) SiO_2_-NPs compared to the control (**a**). Recovery of the signal at 25–100 µg/mL SiO_2_-NPs (**d**–**f**) was detected. In neurons, the signal decreased at 1 µg/mL (**h**) and 100 µg/mL (**l**); on the contrary, at 10–50 µg/mL (**i**–**k**), the signal increased compared to control (**g**). The image is representative of four independent experiments with three technical replicates.

In order to verify our previous results, IQ mapping was performed. This analysis permits the study of large tissue areas in a free-label manner in both visible and IR mode [24,25]. The spectral signal at 989–1185 cm^−1^ that included the SiO_2_-NPs and symmetric PO_2_^−^ of nucleic acid decreased at the concentrations of 1 and 10 µg/mL in astrocytes (Figure S3). The IQ mapping pointed out the low intensity in the spectral signal at the mentioned concentrations, being the lower signal observed at the concentration 10 µg/mL (Figure 5a–c, blue area). However, at concentrations of 25–100 µg/mL, a recovery of the signal was observed; thus, a biphasic effect resulted from SiO_2_-NPs in astrocytes as previously observed (Figure 5d–f, yellow and red areas). In the case of the neurons (Figure S4 and Figure 5g–l), an increase in the spectral signal in the region 989–1185 cm^−1^ was observed at the concentration of 1–25 µg/mL (Figure S4 and Figure 5g–j, indicated in green and red areas). However, this signal decreased at 50 µg/mL (Figure 5k, yellow area), and at the concentration of 100 µg/mL, the lowest signal was detected (Figure 5l, blue area).

### 3.9. IQ Mapping of the Protein Amide I Region 1593–1712 cm^−1^ in Astrocytes and Neurons after Exposure to SiO_2_-NPs

**Figure 6:**
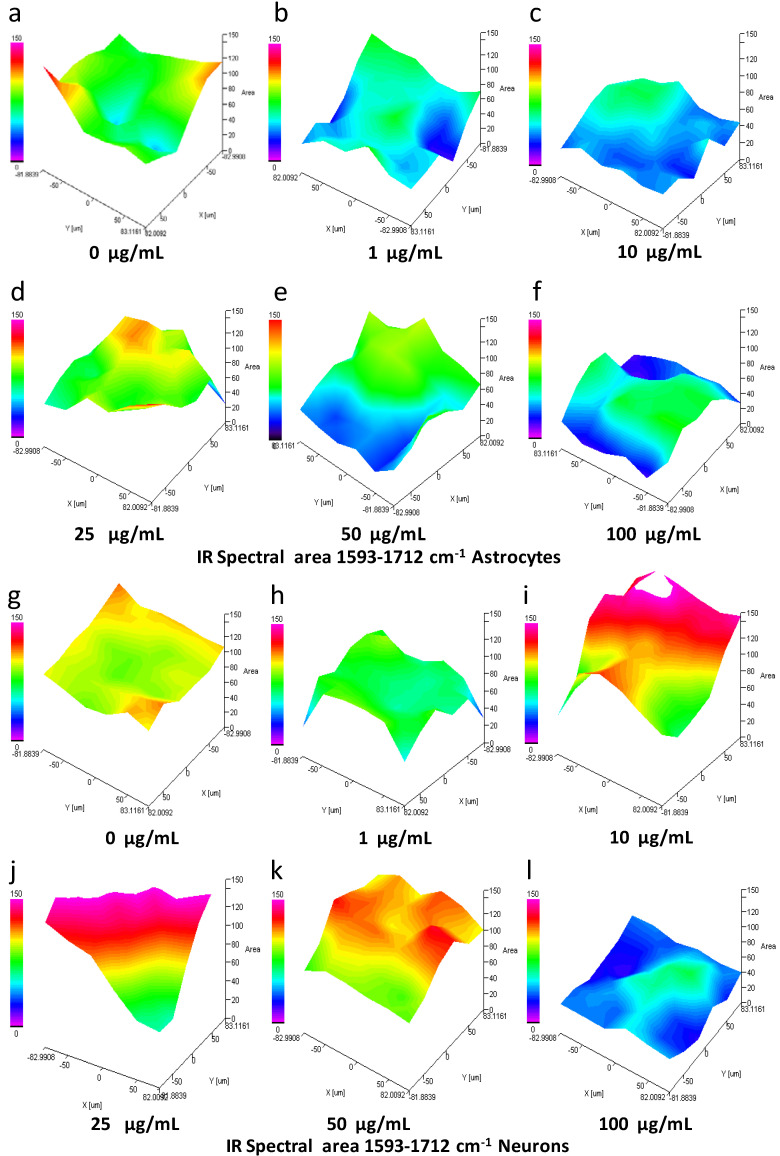
Spectral area analysis by IQ mapping of 1593–1712 cm^−1^ region of astrocytes and neurons exposed to SiO_2_-NPs. The spectral region of 1593–1712 cm^−1^ that includes specific spectral bands related to secondary structures of proteins is presented for astrocytes (**a**–**f**) and neurons (**g**–**l**). A sample of cells treated with SiO_2_-NPs for 24 h was dispersed on a gold-coated slide. The three-dimensional image is presented in the x, y, and z axes, and the area presented as a gradient of color indicates a decrease (from blue) or increase (red) in the spectral signal. In astrocytes, a decrease in the signal seen as a blue area at 1 (**b**), 10 (**c**), 50 (**e**), and 100 (**f**) µg/mL SiO_2_-NPs is observed compared to control (**a**). No changes were observed at 25 µg/mL (d). In neurons, a strong increase in the signal (red area) was detected at 10 (**i**), 25 (**k**), and 50 (**l**) µg/mL. In contrast, at concentration of 1 (**h**) and 100 (**l**) µg/mL, spectral signal decrease (green and blue area) when compared to control (**g**). The image is representative of four independent experiments with three technical replicates.

Analysis by IQ mapping of the protein amide I region 1593–1712 cm^−1^ in rat astrocytes revealed that SiO_2_-NPs cause a substantial decrease in the intensity of the spectral signal; however, this effect did not follow the linear concentration-response pattern (Figure 6a–f). The most noticeable effect occurs at 1 and 10 µg/mL, as indicated in Figure 6b,c (green-blue area). An increase at 25 µg/mL (Figure 6d, yellow area) followed by the decrease at 50-100 µg/mL (Figure 6e,f, blue area) was observed and resemble the biphasic effect previously observed. In contrast, in neurons, SiO_2_-NPs augmented the spectral signal in the region 1593–1712 cm^−1^ at concentrations of 10–50 µg/mL (Figure 6i–k, red area). However, at concentration 100 µg/mL SiO_2_-NPs, the signal decreased (Figure 6l, blue area).

### 3.10. IQ Mapping of the Lipid Region 2830-2945 cm^−1^ in Astrocytes and Neurons after Exposure of SiO_2_-NPs

**Figure 7:**
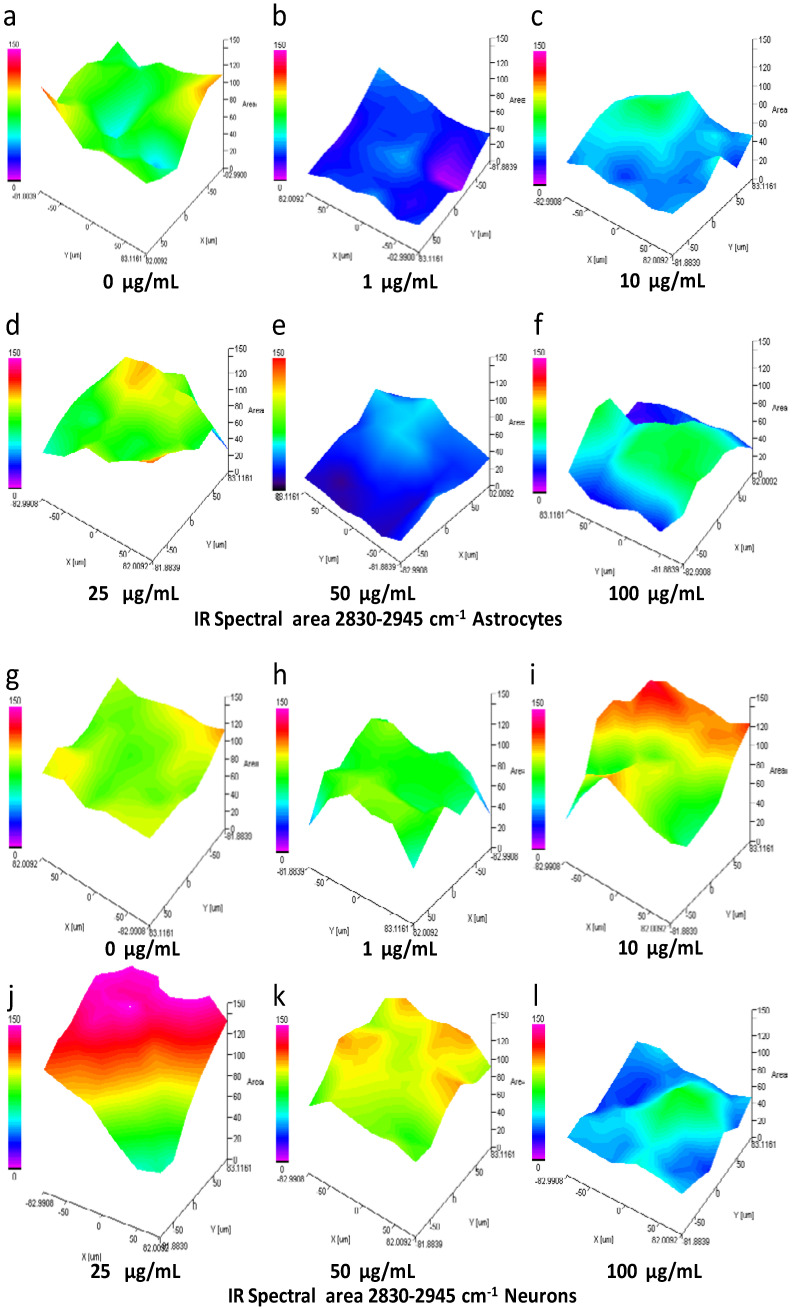
IQ mapping of IR spectral region 2830–2945 cm^−1^ of astrocytes and neurons exposed to SiO_2_-NPs. The spectral region of 2830–2945 cm^−1^ that includes specific spectral bands of symmetric and asymmetric vibrations of -CH_2_ of lipids is presented for astrocytes (**a**–**f**) and neurons (**g**–**l**). A sample of cells treated with SiO_2_-NPs for 24 h was dispersed on a gold-coated slide. The three-dimensional image is presented in the x, y, and z axes, and the area presented as a gradient of color indicates a decrease (from blue) or increase (red) in the spectral signal. In astrocytes (**a**–**f**), a decrease in the signal (blue area) occurred at 1 (**b**), 10 (**c**), 50 (**e**), and 100 (**f**) µg/mL of SiO_2_-NPs when compared to control (**a**). No changes were observed at 25 µg/mL (**d**). In neurons (**g**–**l**)**,** spectral signal increased (red area) from 1–50 µg/mL (**h**–**k**) when compared to control (**g**). The higher increase in signal was detected at 25 µg/mL (**j**). On the contrary, at the concentration of 100 µg/mL (**l**), the lowest spectral signal was detected. The image is representative of four independent experiments with three technical replicates.

Analysis by IQ mapping of the region 2830-2945 cm^−1^ in astrocytes exhibited a decrease in the intensity of the signal in the lipid region that did not follow the linear concentration-response pattern (Figure 7a–f) after exposure to SiO_2_-NPs. The most prominent in the spectral area effect occurs at 1–10 µg/mL (Figure 7b, blue areas), followed by a subtle increase at 25 µg/mL (Figure 7d, green area) and a second decrease at 50–100 µg/mL (Figure 7e,f, blue area). The above agrees with the decrease observed in the ATR-FTIR and second derivative analysis. On the other hand, in neurons at 10–25 µg/mL SiO_2_-NPs, an increase in the spectral area of the region, 2830–2945 cm^−1^ was observed (Figure 7i,j, red area). However, the spectral signal decreased at 50 µ/mL SiO_2_NPs with the lowest signal at 100 µg/mL (Figure 7k,l, yellow, and blue areas, respectively). Collectively, the results from ATR-FTIR spectroscopy and IQ mapping indicated a higher sensitivity of astrocytes to SiO_2_-NPs toxicity than neurons.

### 3.11. Cell Viability Determination

**Figure 8:**
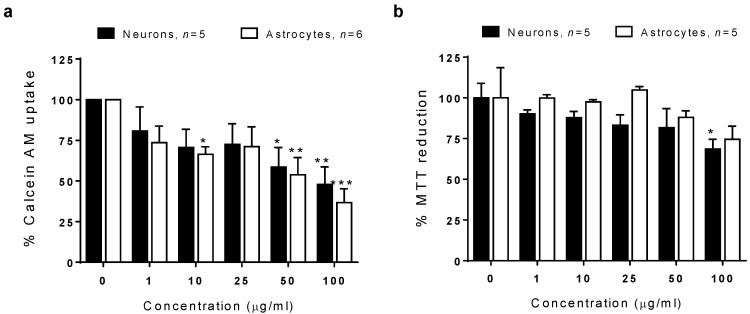
Viability of astrocytes and neurons after exposure to SiO_2_-NPs. The neurons and astrocytes were exposed to 0–100 µg/mL SiO_2_-NPs. (**a**) Calcein-AM uptake in neurons and astrocytes. (**b**) MTT reduction in neurons and astrocytes. Data are presented as mean ± SEM of five to six independent experiments with three technical replicates. Statistical analysis was by two-way ANOVA followed by Bonferroni’s *post hoc* test. *p < 0.05, **p < 0.01, ***p < 0.001 vs. control.

In order to verify the FTIR spectroscopic analysis, we evaluated the effect of SiO_2_-NPs on the cell viability in astrocytes and neurons. The effect of SiO_2_-NPs on the viability evaluated by the calcein-AM uptake assay in astrocytes and neurons resulted in a decrease of 33.4% in the uptake of calcein-AM in astrocytes at a concentration of 10 µg/mL, 46.2% at 50 µg/mL, and 63.3% at 100 µg/mL. In neurons, a significant decrease of 41.4% at 50 µg/mL and 52.2% at 100 µg/mL was detected (Figure 8a). Astrocytes showed a significant decrease in cell viability at a lower concentration than neurons (10 µg/mL), and the significance of the decreases was always higher in astrocytes than those detected in neurons. Therefore, these results confirmed the toxic effects of SiO_2_-NPs on astrocytes and neurons described before and pinpoint the major susceptibility of astrocytes to the toxic effect of SiO_2_-NPs compared to neurons. However, as was previously reported [26], the MTT reduction assay was not useful to estimate the cell death, since we did not detect significant changes (Figure 8b).

## 4. Discussion

In the present study, the toxic effect of SiO_2_-NPs on primary cultures of rat astrocytes and neurons was evaluated by ATR-FTIR spectroscopy and IQ mapping; results showed a higher sensitivity of astrocytes than neurons. The higher susceptibility in astrocytes was evidenced by disturbances in cell morphology and changes in biomolecules, mainly proteins. The adverse effect was associated with changes in biomolecules such as nucleic acids, proteins, and lipids, detectable through alterations in the absorption of specific spectral bands. The principal mechanism of toxicity was related to alterations in secondary structures of proteins in astrocytes. The cell viability test confirmed a higher sensitivity of astrocytes than neurons, and the toxic effect of SiO_2_-NPs was detected since low concentrations.

We obtained spectral bands of pure powder SiO_2_-NPs similar to those reported by Marfin et al. [27]. However, the spectral position of bands of SiO_2_-NPs dispersed in cell culture media was slightly different in the vibrations Si-O out of plane deformation, Si-OH stretching, and Si-O-Si asymmetric stretching. A shift in peak position usually means electron redistribution in the molecular bonds; thus, hydrogen bonding interferences after SiO_2_-NPs dispersion in saline could be a plausible explanation for the shift observed in the peak position. For example, variations in the value of a spectral band have been previously reported in cell culture and could be attributed to residual water present in the sample [28].

Concerning the SiO_2_-NPs physical form, we found an amorphous shape and a size below 100 nm; the hydrodynamic size of SiO_2_-NPs was high because, generally, NPs agglomerates in liquid media. This agglomeration is a crucial aspect of SiO_2_-NPs because their toxic effect depends on size and charge. These two properties are related to the passage of NPs through biological barriers to allocate in the intracellular space and consequently to produce the toxic effect, as was demonstrated with SiO_2_-NPs of 70 nm in a model of intravenous exposure during pregnancy in a mouse model. In that work, the authors found that SiO_2_-NP cross biological barriers and cause alterations in the placenta and damage to the liver and brain in the fetuses [7]. In another example, Kim et al. [29] reported that different sized and charged SiO_2_-NPs would cause differential immunotoxicity, being the small-sized and negatively charged SiO_2_-NPs the most potent immunotoxic NPs. Usually, suspension of any type NPs creates agglomeration, which is bigger than the original size of individual NPs in the dry state. This agglomeration is formed by chemical and/or physical interactions between NPs and the liquid dispersion medium components. Izak-Nau et al. [30] demonstrated that SiO_2_-NPs have spherical form, are core-shell, and are sized 50 nm; they form clusters that remained adsorbed on the surface in cultured astrocytes, followed by a modest absorption. Moreover, the agglomerates or clusters can be internalized and frequently are maintained in vesicles. In some cases, the size of agglomerates in vesicles might be bigger than the cluster before the cell uptake, as has been well demonstrated. For instance, SiO_2_-NPs sized 90 nm showed an agglomeration state of 150 nm in water solution. However, clusters of SiO_2_-NPs from 500 to 1000 nm were detected in vesicles of neural stem cells exposed for 48 h [31]. Clusters of SiO_2_-NPs have been recently detected in endolysosomes of primary cultures of hippocampal neurons from Wistar rats [32]. Importantly, the SiO_2_-NPs uptake in these hippocampal neurons was accompanied by an increase in beta-amyloid levels, which highlights that SiO_2_-NPs internalization might have an impact on some neurodegenerative diseases such as Alzheimer disease.

In our study, there is evidence that the incorporation of the NPs did occur. Uptake of SiO_2_-NPs could be assumed based on the spectral band of 1083 cm^−1^ that corresponds to the most representative band for SiO_2_-NPs with the FTIR analysis. The absorbance of this band increased at all concentrations of SiO_2_-NPs in astrocytes without a change in the control group. We interpreted this as evidence of the uptake of SiO_2_-NPs by astrocytes. Nonetheless, no changes were detected in neurons when we analyzed the same spectral band in the second derivative analysis. Beyond this evidence, the cell uptake of SiO_2_-NPs has been well demonstrated in cell cultures of neural stem cells and hippocampal neurons, but also in other non-brain cell lineages including macrophages [33], intestinal epithelial cell line C2BBe1 [34], keratinocytes [35] and hepatocytes [36].

Namely, SiO_2_-NPs formed agglomerates, and this is a crucial observation because cells can uptake SiO_2_-NPs agglomerates through endocytosis. We must underline that astrocytes and microglia have the phagocytic capacity [37,38,39], and NPs endocytosis has been described mostly in neurons [32]. Once inside the cytoplasm, NPs induce cellular death, as has been demonstrated in vivo and in vitro [8,40].

Moreover, agglomerates of SiO_2_-NPs around 150–200 nm have been detected in primary microglia, and the incorporation is related to phagocytosis. Inside the cell, SiO_2_-NPs may alter microglial function by the increase of ROS and nitrogen oxidative species production, deregulation of pro-inflammatory genes, and cytokine release [41]. As astrocytes are endocytic cells too [42], it is feasible that SiO_2_-NPs incorporation in rat astrocytes and neurons occurred through phagocytosis and endocytosis in the current study. Moreover, as phagocytosis is a highly active mechanism in astrocytes, this could contribute to their higher sensitivity to SiO_2_-NPs toxicity than those observed in neurons. Furthermore, as astrocytes cooperate with neurons at various levels, including trafficking and recycling of neurotransmitters, ionic homeostasis, energy metabolism, and the defense against oxidative stress, any alteration in their function would have a significant impact on the brain. As an example of the effect of NPs on astrocytic function, titanium dioxide NPs induced a significant loss in glutamate uptake associated with mechanisms involved in cell death, like the production of reactive oxygen species and mitochondrial dysfunction [43]. As the glutamate uptake is a fundamental function of astrocytes to avoid excitotoxicity and to provide glutamine to neurons, the nanoparticle exposition could impair synaptic transmission and promote neuronal death. A subject of special attention is the blood–brain barrier (BBB), whose integrity is essential to regulate the movement of molecules between blood and brain. Some reports have revealed significant effects of NPs on BBB integrity. For example, the exposition to silver NPs triggers the increase of permeability in an in vitro model of BBB [44]. In order to understand the effects of SiO_2_-NPs on the modulation of the synaptic transmission in neurons, the effect of SiO_2_-NPs on co-cultures of neurons and astrocytes should be evaluated. Moreover, SiO_2_-NPs interrupted the tight junctions and generated changes in the cytoskeleton in endothelial cells through an IL-6 and VEGF-mediated inflammatory response coordinated with astrocytes [11]. The cytoskeleton alterations reported in the literature and our findings from FTIR spectroscopy about alterations in the secondary structure of proteins encourage us to continue exploring the mechanisms of toxicity and uptake of SIO_2_-NPs in vitro shortly, through the co-culturing of neural and glial cells, infrared spectroscopy, microscopy, and molecular biology.

Regarding the morphological changes observed in astrocytes and neurons, the presence of intracellular NPs would trigger alterations in protein synthesis evidenced through the changes in the absorbance of the protein spectral region observed. In spite that this study was not specifically designed to study the morphological changes in astrocytes and neurons deeply, we realized small morphological changes at low concentrations of SiO_2_-NPs in both cell types and prominent alterations in astrocytes size, cell volume, and loss of branch at the highest concentrations. The above agrees with previous work about morphological changes described in HL-7702 cells after exposure to SiO_2_-NPs for 24 h, where cell volume decreased, together with the presence of cytoplasm and nuclear vacuoles, dense cytoplasm, and formation of apoptotic bodies. At the concentration of 200 µg/mL, apoptosis was detected as nuclear membrane thickening, cell body shrinkage, nuclear pyknosis, and disintegration [45]. In the present study, we observed a biphasic response in astrocytes and neurons after exposure to SiO_2_-NPs. This biphasic behavior has been previously observed with other NPs at low concentrations [46]. For example, in human skin fibroblasts in culture, 0.5 µg/mL SiO_2_-NPs have a beneficial effect through improved survival and function of the cells, meanwhile, at 50 µg/mL or higher, a clear increase of cell death was detected [47]. Although specific molecular mechanisms remain to be fully understood, biphasic responses detected in this study to SiO_2_-NPs support the previous hypothesis in the field, where it has been proposed that biphasic responses could be considered as an emergent physicochemical property of nanoparticles, especially at low concentrations. However, time and environmental exposure scenarios must be included in future studies, as previously reported [46,47].

The alterations detected in spectral regions of nucleic acids, protein, and lipids in astrocytes and neurons are compatible with mechanisms of SiO_2_-NPs toxicity previously reported for other cell types, such as ROS production, lipid oxidation, protein misfolding or reduced synthesis, and cell death by autophagy [48]. These mechanisms have been observed in vitro [48,49,50] and in vivo [8,50] studies. Indeed, morphological changes observed in astrocytes and neurons exposed to SiO_2_-NPs, even at the low concentrations of 1 and 10 µg/mL, may be explained by the ability of SiO_2_-NPs to disrupt proteins, as observed by the changes in the spectral signal of specific bands related to the secondary structure of proteins. In this regard, previous proteomic analysis has evidenced a large number of proteins associated with the cytoskeleton and crucial cellular functions oxidative stress, energy metabolism, apoptosis, among others, disrupted by SiO_2_-NPs [51]. Moreover, silicon materials produce ROS by the interaction between Si and O_2_•- such as SiO•, SiO_2_•, SiO_3_• [50], and considering that unlike neurons, astrocytes contain free most of the complex I and higher ROS production [52], an intracellular oxidant environment could be formed, resulting in more significant damage to DNA and proteins and consequently cell death, as seen for astrocytes in our study.

The decrease in the spectral signal related to the amide I and secondary structures of proteins could be interpreted as a lack of bond formation as a consequence of the protein synthesis decrease or a misfolding of proteins. At similar concentrations, Yang et al. [53] showed that treatment with 10 µg/mL SiO_2_-NPs sized 15 nm for 24 h, induces altered expression of amyloid precursor protein (APP) and neprilysin, enhances phosphorylation of tau, and activates the glycogen synthase kinase (GSK)-3β in human SK-N-SH and rodent neuroblastoma cells. These alterations are related to pathological signs of Alzheimer’s disease; thus, it is likely that neurofilaments and glial filaments have been modified after exposure to SiO_2_-NPs. Additionally, secondary structures of neurofilaments and glial filaments are mainly composed of alpha-helices, and after interaction between ROS and the peptide backbone, those helices could be partly modified into beta-sheet and random coil structures [54]. Hence, the alterations in the secondary structures observed in astrocytes and neurons may be interpreted as a reorganization of the glial filament and neurofilament structure after interaction with ROS formed as a consequence of SiO_2_-NPs exposure. The above is a plausible explanation for the astrocytic and neuronal degeneration observed after exposure to SiO_2_-NPs in this study.

Although the increase in the absorbance of spectral bands related to lipids due to SiO_2_-NPs was not statistically different in neurons, it cannot rule out that SiO_2_-NPs has a deleterious effect in the lipid membrane promoting neuronal death at higher concentrations as in the case of astrocytes. Meanwhile, autophagy could be involved in astrocytes’ cell death; it is possible that in neurons, apoptosis/necrosis could be the mechanism of cell death implied. The above is supported by the increase in absorbance at 2852 cm^−1^ and 2923 cm^−1^ observed in this study, which agrees with an increase in the same spectral bands reported in U937 and CCRF-CEM cells that underwent to apoptosis/necrosis after several acute stress conditions and were studied by FTIR spectroscopy [22]. In contrast, PC12 cells exposed directly to SiO_2_-NPs size 25 nm at 25–200 µg/mL for 24 h, increased NPs uptake, and autophagy in a concentration-dependent way [55]. These discrepancies highlight the need for more studies to explain the mechanisms and factors involved in neural death after acute exposure to SiO_2_-NPs.

Finally, we found that ATR-FTIR spectroscopy together with IQ mapping was useful to evaluate the toxic effect of SiO_2_-NPs and also allowed the study of the molecular fingerprint after exposure to NPs avoiding the need of reagents or complicated manipulation of the samples [14,22]. However, one of the limitations of the FTIR analysis is that we cannot distinguish the groups of affected proteins, but this technique performed a rapid screening of cell status after exposure to toxic substances. Moreover, the second derivative analysis enabled the elucidation of the mechanisms involved in the decrease of viability after exposure to the NPs. The cell viability test based on Calcein-AM uptake was an excellent tool to evaluate cell viability and confirmed the higher susceptibility of astrocytes to the toxic effect of SiO_2_-NPs compared to neurons, but MTT reduction was not the best way to evaluate cell viability of astrocytes. The above observation agrees with a previous report that discourages the use of MTT to evaluate nanoparticle toxicity [26] since mesoporous silicon NPs stimulate the formazan exocytosis, rendering an overestimation of the cytotoxicity. In spite that we found the contrary effect, ergo an underestimation of cytotoxicity using MTT reduction, our results using SiO_2_-NPs that are not mesoporous confirmed that MTT reduction should not be used for silicon nanoparticle cytotoxicity evaluation. The above could be related with the mainly glycolytic metabolism that is present in astrocytes. MTT reduction is a method based on the enzymatic activity of cell dehydrogenases like lactate dehydrogenase (which converts pyruvate to lactate) or succinate dehydrogenase (an enzyme from oxidative metabolism).

In conclusion, our data show that primary cultures of astrocytes displayed higher sensitivity than neurons to SiO_2_-NPs toxicity, evidenced through morphological changes, alterations in the IR spectral signal of nucleic acids, lipids, and proteins. The main mechanisms involved in the toxicity of SiO_2_-NPs, specifically for astrocytes, involves alterations in the secondary structure of proteins. However, additional studies are necessary to deepen the knowledge about neurons and astrocytes susceptibility to toxic stimuli. Moreover, complementary studies exploring the effects of SiO_2_-NPs to evaluate the interaction between glia and neurons could be performed to enlarge the precise mechanism involved in cell death.

## Figures and Tables

**Table 1:** Second derivative analysis of spectral bands (cm^−1^) of chemical bond for nucleic acids, protein and lipid regions of rat astrocytes and neurons exposed to SiO2-NPs.

Cell Type/Chemical Bond	Concentration of SiO_2_-NPs (µg/mL)							
		0	1	10	25	50	100	
**Astrocytes**								
**Vibration**	**Spectral Band**	***n*** **= 8**	***n*** **= 8**	***n*** **= 8**	***n*** **= 8**	***n*** **= 8**	***n*** **= 8**	**ANOVA**
								***P***
SiO_2_-NPs	1083 cm^−1^	0.023 ± 0.010	0.019 ± 0.008	0.014 ± 0.003 ^§^	0.017 ± 0.006	0.020 ± 0.006	0.028 ± 0.011	0.0177 *
Symmetric PO_2_^−^	1090 cm^−1^	0.024 ± 0.011	0.019 ± 0.008	0.013 ± 0.003 ^§^	0.017 ± 0.006	0.020 ± 0.007	0.027 ± 0.012	0.0211 *
Parallel beta strand	1627 cm^−1^	0.026 ± 0.010	0.021 ± 0.008	0.015 ± 0.003 ^§^	0.019 ± 0.005	0.022 ± 0.006	0.029 ± 0.011	0.0167 *
Random coil	1639 cm^−1^	0.031 ± 0.012	0.026 ± 0.008	0.019 ± 0.003 ^§^	0.023 ± 0.005	0.025 ± 0.007	0.033 ± 0.011	0.0241 *
Alpha Helix	1654 cm^−1^	0.034 ± 0.012	0.030 ± 0.009	0.022 ± 0.003 ^§^	0.026 ± 0.005	0.029 ± 0.008	0.036 ± 0.010	0.0246 *
Symmetric -CH_2_	2852 cm^−1^	0.029 ± 0.010	0.026 ± 0.009	0.019 ± 0.003 ^§^	0.025 ± 0.004	0.026 ± 0.008	0.033 ± 0.011	0.0452 *
Asymmetric -CH_2_	2923 cm^−1^	0.029 ± 0.010	0.025 ± 0.008	0.019 ± 0.003 ^§^	0.024 ± 0.004	0.026 ± 0.008	0.032 ± 0.011	0.0292 *
**Neurons**								
**Vibration**	**Spectral Band**	***n*** **= 11**	***n*** **= 11**	***n*** **= 11**	***n*** **= 11**	***n*** **= 11**	***n*** **= 11**	**ANOVA**
								***P***
SiO_2_-NPs	1083 cm^−1^	0.012 ± 0.003	0.014 ± 0.006	0.014 ± 0.004	0.012 ± 0.003	0.013 ± 0.004	0.015 ± 0.007	0.5955
Symmetric PO_2_^−^	1090 cm^−1^	0.012 ± 0.003	0.014 ± 0.007	0.014 ± 0.004	0.013 ± 0.003	0.014 ± 0.004	0.016 ± 0.007	0.5404
Parallel beta strand	1627 cm^−1^	0.015 ± 0.003	0.018 ± 0.006	0.018 ± 0.004	0.016 ± 0.003	0.017 ± 0.003	0.019 ± 0.007	0.4314
Random coil	1639 cm^−1^	0.016 ± 0.003	0.019 ± 0.008	0.019 ± 0.005	0.017 ± 0.003	0.018 ± 0.00	0.020 ± 0.007	0.5086
Alpha Helix	1654 cm^−1^	0.021 ± 0.004	0.024 ± 0.008	0.024 ± 0.005	0.028 ± 0.003	0.024 ± 0.004	0.027 ± 0.008	0.3475
Symmetric -CH_2_	2852 cm^−1^	0.028 ± 0.003	0.030 ± 0.007	0.031 ± 0.004	0.030 ± 0.003	0.031 ± 0.004	0.033 ± 0.008	0.4128
Asymmetric -CH_2_	2923 cm^−1^	0.026 ± 0.003	0.028 ± 0.007	0.029 ± 0.004	0.028 ± 0.003	0.029 ± 0.004	0.030 ± 0.008	0.4027

* One-way ANOVA indicates difference among groups, *p* < 0.05; § Difference between SiO_2_-NPs concentration 10 vs. 100 µg/mL, Tukey’s *post hoc* test, *p* < 0.05.

## Supplementary Materials

The following are available online at https://www.mdpi.com/2305-6304/8/3/51/s1, Table S1: Spectral bands (cm^−1^) of chemical bonds of biomolecules detected by ATR-FTIR Spectroscopy in rat astrocytes and neurons exposed to SiO_2_-NPs; Figure S1. Identification of cell cultures; Figure S2. A spectral fingerprint of rat astrocytes and neurons exposed to SiO_2_-NPs; Figure S3. FTIR microscopy and IQ mapping analysis of the spectral region of 989–1185 cm^−1^ of astrocytes exposed to SiO_2_-NPs; Figure S4. FTIR microscopy and IQ mapping analysis of the spectral region of 989–1185 cm^−1^ of astrocytes exposed to SiO_2_-NPs. FTIR microscopy analysis and IQ mapping of neurons exposed to SiO_2_-NPs.
