# Supplementary material for: Astrocytes Are More Vulnerable than Neurons to Silicon Dioxide Nanoparticle Toxicity in Vitro
Source: Toxics. 2020 Jul 29;8(3):51. doi: 10.3390/toxics8030051 (PMC7560395; doi:10.3390/toxics8030051)
Supplement: Supplementary file 1 [file toxics-08-00051-s001.pdf]

toxics

Supplementary Materials: Astrocytes are More Vulnerable than Neurons to Silicon Dioxide Nanoparticle Toxicity in Vitro

Jorge Humberto Limón-Pacheco, Natalie Jiménez-Barrios, Alejandro Déciga-Alcaraz, Adriana Martínez-Cuazitl, Mónica Maribel Mata-Miranda, Gustavo Jesús Vázquez-Zapién, Jose Pedraza-Chaverri [5], Yolanda Irasema Chirino and Marisol Orozco-Ibarra

Table S1. Spectral bands (cm^{-1}) of chemical bonds of biomolecules detected by ATR-FTIR Spectroscopy in rat astrocytes and neurons exposed to SiO_2-NPs.

Cell Type	Chemical Bonds (Vibration)	Concentration of SiO_2NPs					
		0 mg/mL	1 mg/mL	10 mg/mL	25 mg/mL	50 mg/mL	100 mg/mL
		Spectral bands wavenumber cm^{-1}					
Astrocytes							
	-OH stretching	3293	3294	3296	3297	3299	3302
	Asymmetric $-CH_2$ acyl chains (lipids)	2926	2924	2924	2924	2925	2925
	Symmetric $-CH_2$ (lipids)	2855	2853	2853	2853	2856	2855
	C=O (lipids)	1744	1742	1735	1740	1739	1740
	Amide I (mainly protein C=O stretching)	1650	1654	1653	1654	1652	1653
	Amide II (protein N–H bending, C–N stretching)	1545	1545	1545	1545	1546	1545
	Asymmetric PO_2^- (nucleic acid)	1237	1240	1239	1237	1241	1230
	Symmetric PO_2^- (nucleic acid)	1090	1087	1086	1086	1088	1089
Neurons							
	-OH stretching	3290	3291	3290	3290	3290	3290
	Asymmetric $-CH_2$ acyl chains (lipids)	2923	2923	2923	2923	2923	2923
	Symmetric $-CH_2$ (lipids)	2852	2852	2852	2852	2852	2852
	C=O (lipids)	1740	1741	1741	1741	1741	1740

**Amide I (mainly protein C=O stretching)**	1652	1653	1652	1652	1652	1653
**Amide II (protein N–H bending, C–N stretching)**	1545	1544	1544	1544	1544	1544
**Asymmetric PO_2^- (nucleic acid)**	1237	1237	1237	1237	1237	1236

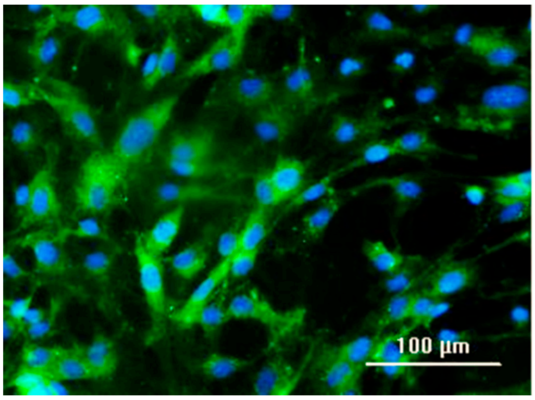

(a)

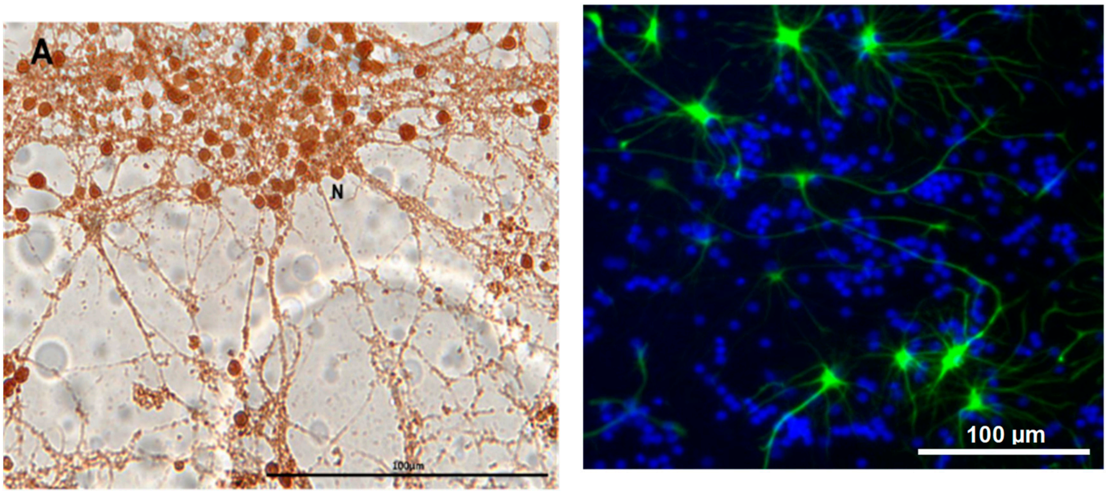

(b)

Figure S1. Identification of cell cultures. Immunostaining of astrocyte-rich secondary cultures (**a**) where glial fibrillary acidic protein (GFAP) is shown in green and 4′,6-diamidino-2-phenylindole (DAPI) is shown in blue for nuclei detection. As expected, most of the cells were astrocytes, as they express GFAP. Immunostaining of cerebellar granule neuron-rich cultures (**b**), the left micrograph shows microtubule-associated protein 2 (MAP-2) by immunocytochemistry; the right micrograph shows GFAP in green and DAPI in blue by immunofluorescence. We used the MAP-2 stain to prove that most of the cells were neurons, and the DAPI stain to count the total number of neurons. The GFAP stain exposed the glial cells that are present in the cultures. Scale bars: 100 µm.

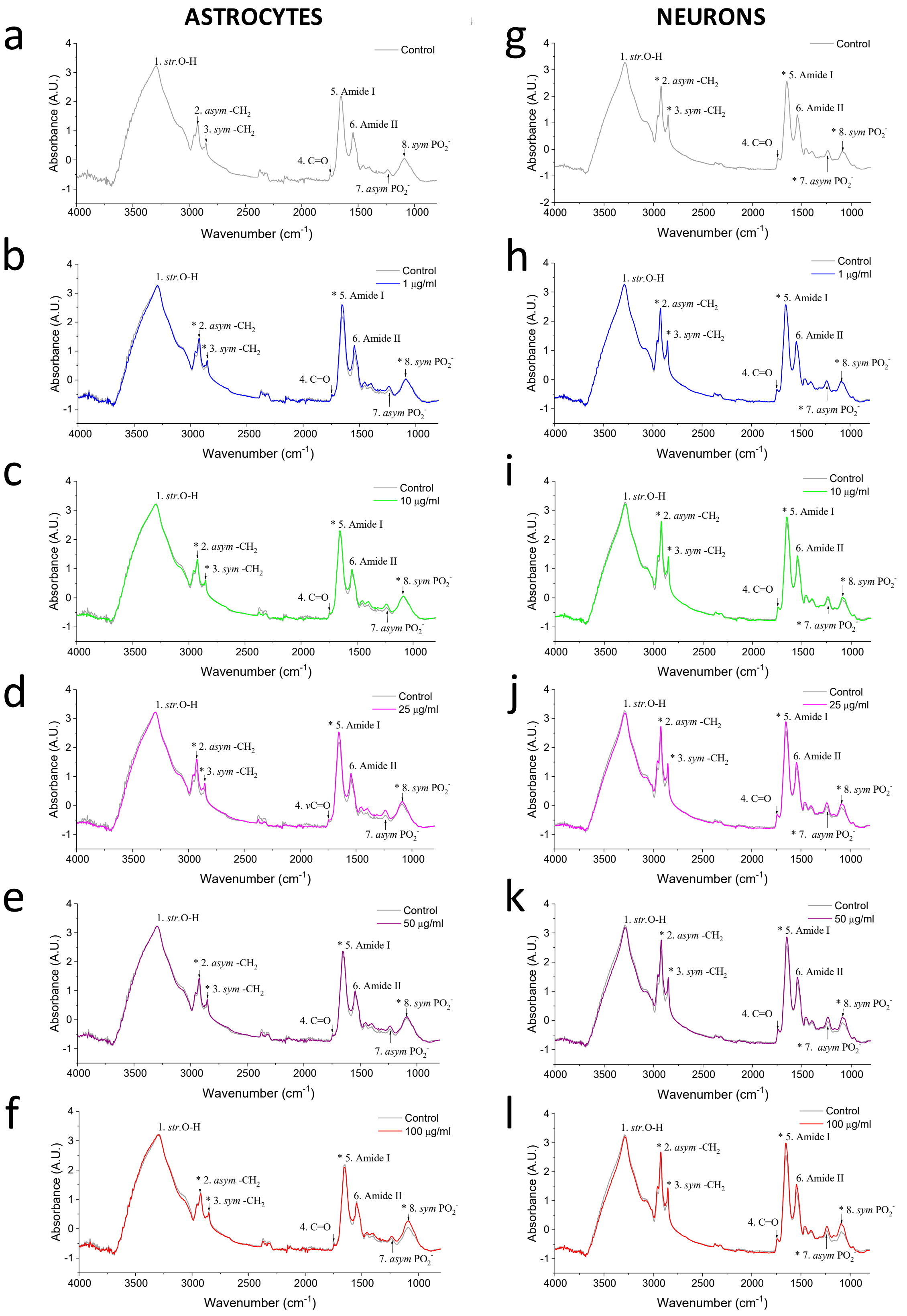

Figure S2. A spectral fingerprint of rat astrocytes and neurons exposed to SiO_2-NPs. A sample of four µL of cells treated with SiO_2-NPs for 24 h was placed onto the surface of the ATR crystal, as was described in the material and methods section. The numbers correspond to different spectral bands of chemical bonds of biomolecules of the fingerprint of the toxicity of SiO_2-NPs in astrocytes (**a**–**f**) and neurons (**g**–**l**). For astrocytes, spectral bands for lipids were (1) -OH stretching at 3293-3302 cm^{-1}, (2) asymmetric $-CH_2$ acyl chains at 2924–2926 cm^{-1}, (3) symmetric $-CH_2$ at 2853–2855 cm^{-1}, and (4) C=O at 1739–1744 cm^{-1}. Spectral bands of protein region: (5) protein C=O stretching of amide I at 1650–1654

cm^{-1}, and (6) protein N–H bending and C–N stretching of amide II at 1545–1546 cm^{-1}. Spectral bands for nucleic acids region: (7) asymmetric PO_2^- at 1230–1241 cm^{-1}, (8) symmetric PO_2^- at 1086–1090 cm^{-1}. For neurons (g–l), spectral bands for lipids were (1) -OH stretching at 3290–3291 cm^{-1}, (2) asymmetric -CH_2 acyl chains at 2923 cm^{-1}, (3) symmetric -CH_2 at 2852 cm^{-1}, and (4) C=O at 1740–1741 cm^{-1}. Spectral bands of protein region: (5) protein C=O stretching of amide I at 1652–1653 cm^{-1}, and (6) protein N–H bending and C–N stretching of amide II at 1544–1545 cm^{-1}. Spectral bands for nucleic acids region: (7) asymmetric PO_2^- at 1236–1237 cm^{-1}, (8) symmetric PO_2^- at 1088–1090 cm^{-1}. The image is representative of four independent experiments with three technical replicates.

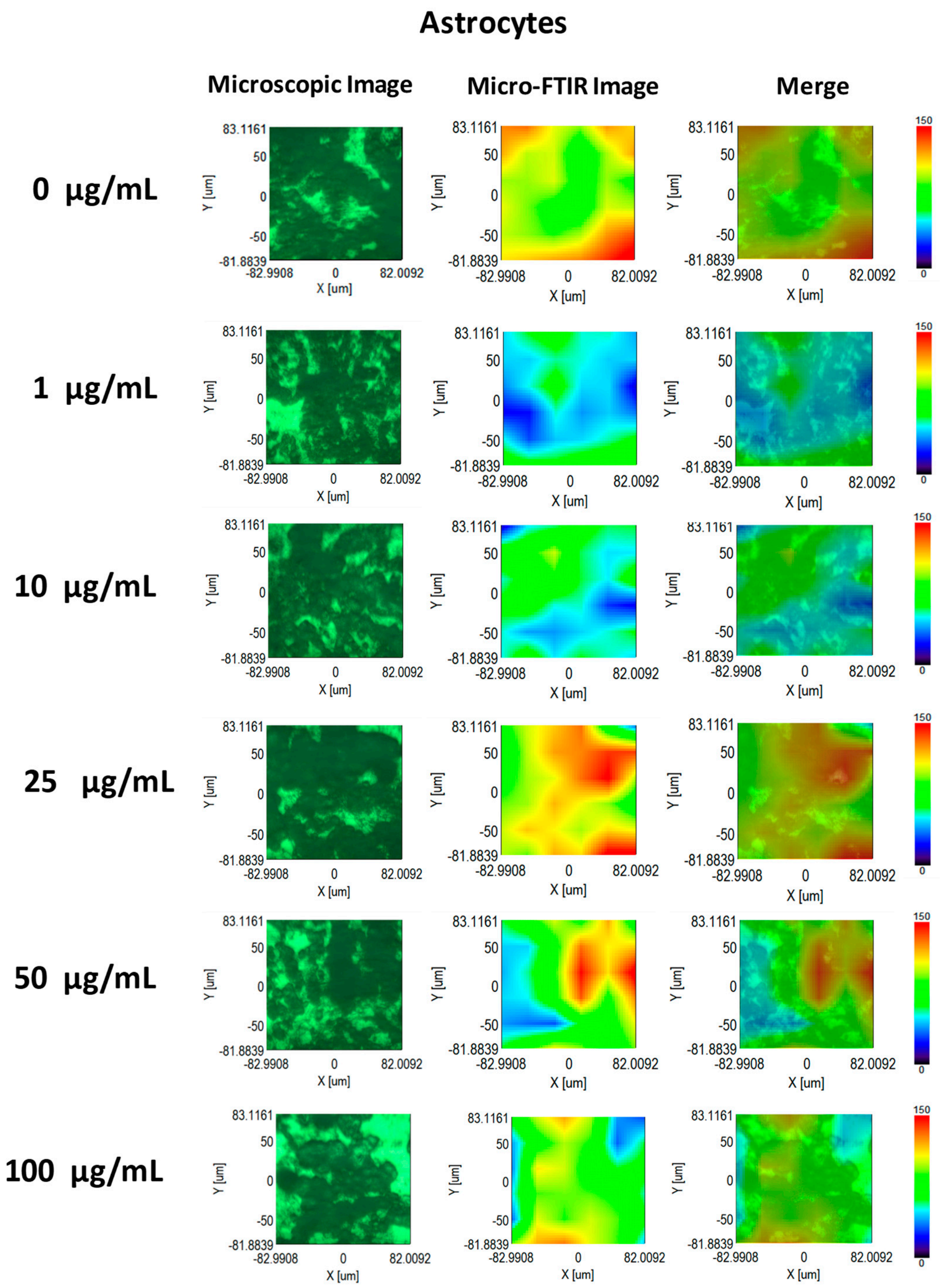

Figure S3. FTIR microscopy and IQ mapping analysis of the spectral region of 989–1185 cm^{-1} of astrocytes exposed to SiO_2-NPs. From left to right, in the first column, the concentrations of SiO_2-NPs are indicated, in the second column, a representative image of the astrocytes from each experimental concentration observed in the FTIR microscope in bright field mode is shown. The third column

shows the images using the IQ mapping for the spectral region of 989–1185 cm⁻¹, which includes the spectral band 1090 cm⁻¹ of the symmetric PO_2^- DNA and the 1083 cm⁻¹ band that represents the asymmetric stretching Si-O-Si vibration. Merge of the bright field, and IQ mapping mode is shown in the fourth (right) column, and a decrease in the signal can be seen at concentrations of 1, and 10 µg/mL SiO_2-NPs and recovery in at 25–100 µg/mL similar to control (right column) is observed. A representative image of 4 independent experiments performed in duplicate for each concentration is shown.

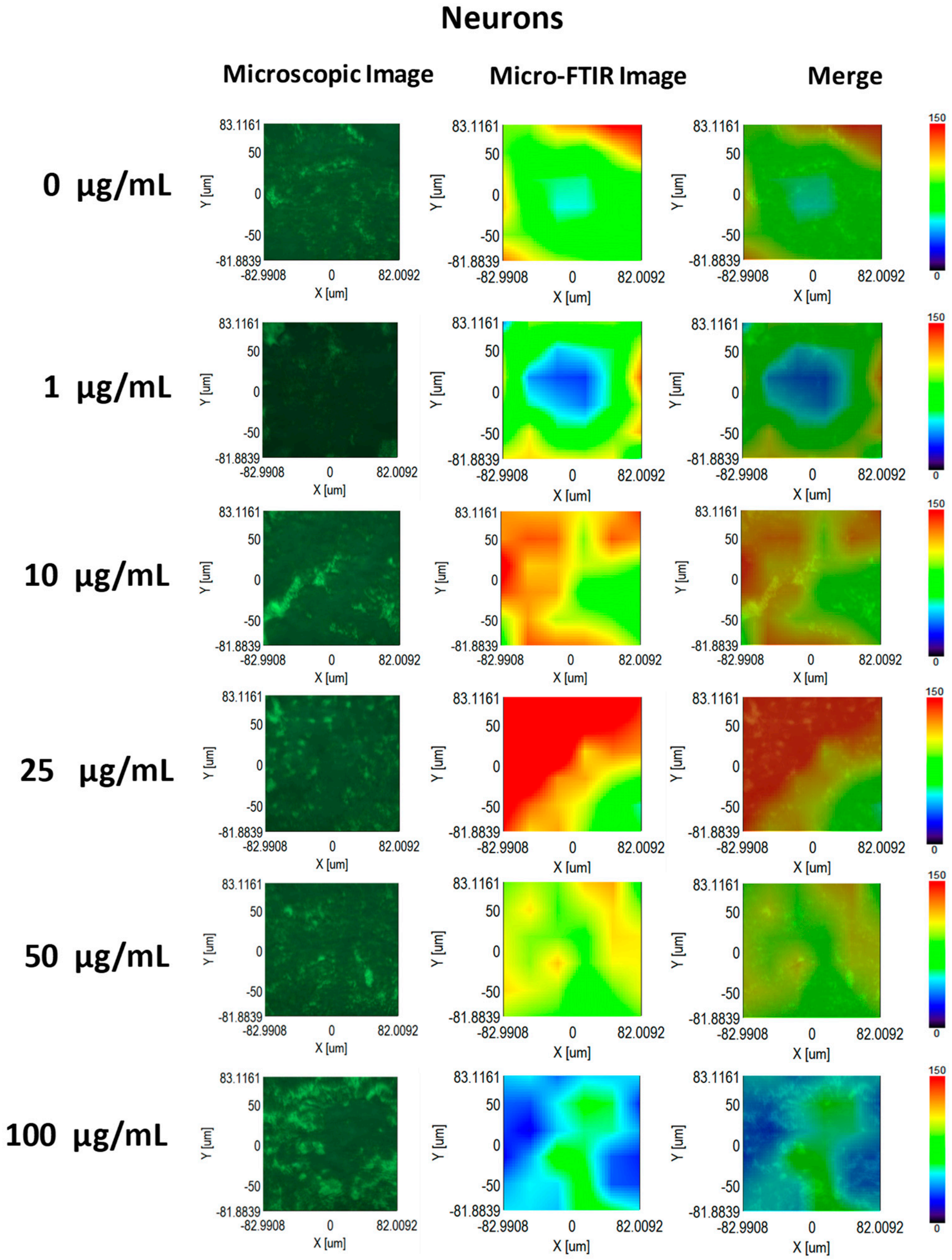

Figure S4. FTIR microscopy and IQ mapping analysis of the spectral region of 989–1185 cm⁻¹ of astrocytes exposed to SiO_2-NPs. FTIR microscopy analysis and IQ mapping of neurons exposed to SiO_2-NPs. From left to right, in the first column, the concentrations of SiO_2-NPs are indicated; in the second column, a representative image of neurons observed under the FTIR microscope in bright field mode is shown. The third column shows the image using the IQ mapping mode for the spectral region of 989–1185 cm⁻¹, which includes the spectral band 1090 cm⁻¹ of the symmetric PO_2^- DNA and the 1083 cm⁻¹ band of the asymmetric stretching Si-O-Si vibration. Merge showed a slight increase in the

spectral signal at the concentrations of 25 and 50 μg/mL SiO_2-NPs (right column). The image is representative of 4 independent experiments performed in duplicate.
